# Conserved oligomeric Golgi (COG) complex genes functioning in defense are expressed in root cells undergoing a defense response to a pathogenic infection and exhibit regulation my MAPKs

**DOI:** 10.1371/journal.pone.0256472

**Published:** 2021-08-26

**Authors:** Vincent P. Klink, Omar Darwish, Nadim W. Alkharouf, Bisho R. Lawaju, Rishi Khatri, Kathy S. Lawrence

**Affiliations:** 1 USDA ARS NEA BARC Molecular Plant Pathology Laboratory, Beltsville, MD, United States of America; 2 Department of Mathematics Computer Science, Texas Woman’s University, Denton, TX, United States of America; 3 Department of Computer and Information Sciences, Towson University, Towson, MD, United States of America; 4 Department of Entomology and Plant Pathology, Auburn University, Auburn, AL, United States of America; 5 Department of Biological Sciences, Mississippi State University, Mississippi, MS, United States of America; Texas Tech University, UNITED STATES

## Abstract

The conserved oligomeric Golgi (COG) complex maintains correct Golgi structure and function during retrograde trafficking. *Glycine max* has 2 paralogs of each COG gene, with one paralog of each gene family having a defense function to the parasitic nematode *Heterodera glycines*. Experiments presented here show *G*. *max* COG paralogs functioning in defense are expressed specifically in the root cells (syncytia) undergoing the defense response. The expressed defense COG gene COG7-2-b is an alternate splice variant, indicating specific COG variants are important to defense. Transcriptomic experiments examining RNA isolated from COG overexpressing and RNAi roots show some COG genes co-regulate the expression of other COG complex genes. Examining signaling events responsible for COG expression, transcriptomic experiments probing MAPK overexpressing roots show their expression influences the relative transcript abundance of COG genes as compared to controls. COG complex paralogs are shown to be found in plants that are agriculturally relevant on a world-wide scale including *Manihot esculenta*, *Zea mays*, *Oryza sativa*, *Triticum aestivum*, *Hordeum vulgare*, S*orghum bicolor*, *Brassica rapa*, *Elaes guineensis* and *Saccharum officinalis* and in additional crops significant to U.S. agriculture including *Beta vulgaris*, *Solanum tuberosum*, *Solanum lycopersicum* and *Gossypium hirsutum*. The analyses provide basic information on COG complex biology, including the coregulation of some COG genes and that MAPKs functioning in defense influence their expression. Furthermore, it appears in *G*. *max* and likely other crops that some level of neofunctionalization of the duplicated genes is occurring. The analysis has identified important avenues for future research broadly in plants.

## Introduction

The conserved oligomeric Golgi (COG) complex maintains the correct Golgi apparatus structure as well as function with a role in retrograde trafficking in eukaryotes [1]. The COG complex performs functions in homeostasis, in particular, regarding protein glycosylation [1, 2]. By virtue of their role in retrograde trafficking, the COG complex has a central cellular role, broadly, in eukaryotes.

The COG complex is composed of 8 subunits that coalesce into A and B sub-complexes [1, 3–6]. The A sub-complex is composed of COGs1-4 while the B sub-complex is composed of COGs5-8 [1, 7]. Notably, COG complex components interact with other proteins including the soluble N-ethylmaleimide-sensitive factor attachment protein receptor (SNARE) which is a major component of the cellular membrane fusion apparatus [4, 8–10]. The COG complex functions with several other associated proteins, including Rabs, various tethers containing coiled-coil proteins, as well as molecular motors which facilitate its many functions [4, 9]. Due to its retrograde trafficking role, the COG complex performs a central function in the delivery of materials between the Golgi cisternae.

The initial understanding of the COG complex came from genetic studies made in the experimental model *Saccharomyces cerevisiae* [3, 11, 12]. The experiments revealed the growth deficiencies of mutants came from Golgi complex impairment involving retrograde trafficking [3, 11, 12]. COG complex genes have also been identified in humans, with their mutations causing various types of disease and growth defects [13–15]. In plants, experiments examining the genetic model *Arabidopsis thaliana* show COG7 mutations impair cell expansion and meristem organization [16]. While much information has been obtained for COG genes in human and *S*. *cerevisiae*, very little is known regarding the COG complex in plants with even less understood regarding their role during their pathogenic interactions.

Recent experiments focusing in on COG complex biology occurring during plant pathogenic interactions have been performed on *G*. *max* infected with the parasitic nematode *Heterodera glycines* (soybean cyst nematode [SCN]) [17]. *H*. *glycines* is the most economically important pathogen of *G*. *max*, accounting for a 7–10% decrease in yield while causing more economic loss than the rest of its pathogens combined so any knowledge on defense is urgently needed [18, 19]. More broadly, information in this pathosystem can aid in understanding plant defense mechanisms in other pathosystems [20]. *G*. *max* may show clear signs of *H*. *glycines* activity, including stunting and even chlorosis. However, in some cases no adverse signs of parasitism are evident, except a decrease in yield of approximately 15% [21].

The *H*. *glycines* life cycle has a 30-day duration, dependent on ambient temperatures [22]. The life cycle of *H*. *glycines* begins as a hardened female carcass (cyst) containing 250–500 fertilized eggs, present within the soil and may remain dormant for up to 9 years [22]. Proper conditions lead to egg hatch, liberating second stage juveniles (J2s) which migrate toward and subsequently burrow into the root, slicing through root cells with a rigid, tubular mouth apparatus known as a stylet. This process takes approximately 24 hours for the J2 to reach its site of parasitism [23, 24]. The *H*. *glycines* stylet then is used to deliver effectors into a *G*. *max* pericycle or neighboring cell that it will parasitize. The *H*. *glycines*-parasitized root cell walls then dissolve. The cell walls dissolve due to processes facilitated by the nematode, resulting in 200–250 neighboring root cells becoming incorporated into a common cytoplasm producing a syncytium. The syncytium is also the site of the localized defense responses, driven by plant-mediated processes that include pathogen activated molecular pattern (PAMP) triggered immunity (PTI) and effector triggered immunity (ETI) [17, 23–32]. The original *H*. *glycines* resistance loci identified in this plant-pathosystem include the recessive *rhg1*, *rhg2* and *rhg3* and the dominant *Rhg4* and *Rhg5* [33–35]. The *rhg1* locus is the most effective at combating *H*. *glycines* parasitism, containing copies of tandemly repeated gene-containing cassettes composed of an amino acid transporter, a wound inducible protein and a membrane fusion protein known as alpha soluble N-ethylmaleimide-sensitive fusion protein (α-SNAP) [28, 36]. The α-SNAP gene has a role in resistance in *G*. *max* to *H*. *glycines* which is in agreement with observations of how membrane fusion functions in the plant defense to pathogens [29, 36–40]. To further exemplify the importance of membrane trafficking to plant defense to pathogens, in particular retrograde trafficking, experiments performed on *Hordeum vulgare* (wheat) identified a defense role for COG3 (*Hv*COG3) to fungal infection by *Blumeria graminis* f.sp. hordei [41]. The result indicated that the COG complex may function broadly in defense across different plant species to different pathogens. To determine a defense role for the *G*. *max* COG complex, a genomic analysis using *S*. *cerevisiae* COG protein sequences led to the identification of 2 paralogs for each COG gene. Functional studies demonstrated that one of the two paralogs of each COG gene family function in the defense process [17]. Furthermore, seed treatment with the bacterial effector harpin that functions in ETI leads to the induced transcript abundance of COG paralogs that function in the defense process [17]. Experiments have shown the syntaxin 31 homolog of *S*. *cerevisiae*, suppressors of the erd2-deletion 5 (Sed5p) which is a SNARE component, binds to Sec17p, COG4 and COG6 [42–46]. The *G*. *max* syntaxin 31 homolog, SYP38, functions in the defense process to *H*. *glycines* with its overexpression co-regulating α-SNAP-5 expression [30]. Lawaju et al. (2020) took those experiments further, showing an increased syntaxin 31 transcript level in each of the transgenic COG overexpressing roots that impair *H*. *glycines* parasitism [17].

A shortcoming in the experiments of Lawaju et al. (2020) [17] was the lack of a demonstration of whether any of the COG genes that function in the defense process are actually expressed within the root cells that are parasitized by *H*. *glycines*. This knowledge is important because syncytium-transcription has been an important trait in identifying genes functioning in the defense process that *G*. *max* has toward *H*. *glycines* [28, 29, 32, 47]. In the experiments presented here, transcriptomic data is presented showing the COG gene expression occurring within *H*. *glycines*-parasitized root cells undergoing a defense response in the *G*. *max*_[Peking/PI 548402]_ and *G*. *max*_[PI 88788]_ genotypes that are capable of a defense response to *H*. *glycines*. The newly presented data strengthens the functional transgenic data of Lawaju et al. (2020) [17] obtained from COG overexpressing and RNAi roots by showing that the COG paralogs that are expressed within the syncytia undergoing a defense response are those that function in the defense response. Importantly, experiments reveal that one of those genes is a splice variant of a COG complex gene (COG7-2, Glyma.12G013000.2 [COG7-2-b]), other than its primary transcript (Glyma.12G013000.1 [COG7-2-a]), is expressed within the syncytium undergoing the defense response and functions in the defense process. To obtain a basic understanding of *G*. *max* COG gene expression, RNA seq data has been extracted from Phytozome and analyzed to examine the relative transcript abundance of these COG complex gene splice variants in RNA samples from leaf, nodule, pod, root, root hair, seed shoot apical meristem (SAM) and stem examined in biological triplicate using Phytomine [48–50]. Due to the functionality of the *Hordeum vulgare Hv*COG3 in defense, COG complex gene family member gene sequence data have been extracted from various genome sources, including their alternate splice variants, from a number of important crop plants [41]. In some cases, extensive numbers of splice variants have been identified from the genomes of these plant species. Prior experiments in the *G*. *max-H*. *glycines* pathosystem have identified the co-regulation of components of the vesicle transport apparatus and mitogen activated protein kinase (MAPK) signaling. Experiments presented here show COG overexpressing and RNAi roots exhibit COG gene co-regulation. Furthermore, prior experiments have shown the specific *G*. *max* MAPK genes function during its defense response to *H*. *glycines* parasitism [32]. To gain an understanding of signaling processes relating to the *G*. *max* COG complex expression, RNA seq analyses, followed by RT-qPCR experiments, determine whether the relative transcript abundance of COG genes becomes affected by MAPK gene overexpression [51].

## Materials and methods

### COG component gene identification

The genome sequences, assemblies and annotations for *A*. *thaliana*, *G*. *max*, *M*. *esculenta*, *Z*. *mays*, *O*. *sativa*, *T*. *aestivum*, *H*. *vulgare*, *S*. *bicolor*, *B*. *rapa*, *S*. *tuberosum*, *S*. *lycopersicum* and *G*. *hirsutum* are housed at Phytozome (https://phytozome.jgi.doe.gov) [49, 52–69]. The genome sequences for *E*. *guineensis* (http://gbrowse.mpob.gov.my)*; S*. *officinalis* (https://sugarcane-genome.cirad.fr/ and *B*. *vulgaris* (https://bvseq.boku.ac.at/) have also been mined [59, 70–72]. The proteomes have been queried with the conceptually translated *A*. *thaliana* COG gene sequences using Arabidopsis thaliana TAIR10. This process has been performed using the Basic Local Alignment Search Tool (BLAST) [73]. Some queries have been performed in Phytozome [49]. In those cases, the default settings have been used. The default parameters include: Target type: Proteome; Program: BLASTP-protein query (BLASTP 2.2.26+) to protein database; Expect (E) threshold: -1; Comparison matrix: BLOSUM62; Word (W) length: default = 3; number of alignments to show: 100 allowing for gaps and filter query. The proteomes analyzed at Phytozome (https://phytozome.jgi.doe.gov) have included *Glycine max Wm82*.*a2*.*v1* (soybean), *Manihot esculenta v6*.*1* (cassava), *Zea mays Ensembl-18* (Maize), *Oryza sativa v7_JGI* (rice), *Triticum aestivum v2*.*2* (common wheat), *Hordeum vulgare r1* (barley), *Sorghum bicolor v3*.*1*.*1* (Cereal grass), *Brassica rapa FPsc v1*.*3* (Turnip mustard–FasPlant), *Solanum lycopersicum iTAG2*.*4* (Tomato), *Solanum tuberosum v4*.*03* (Potato), *Gossypium hirsutum v1*.*1* (Upland cotton) [53, 55–57, 61–66, 69, 74–76]. Additional proteomes for *E*. *guineensis; S*. *officinalis* and *B*. *vulgaris* have also been mined [59, 70, 71]. The oil palm genome has been mined using E. guineensis Genes.faa (v3) employing BLOSUM62 under their default settings [70]. The sugarcane R570 cultivar genome has been mined using BLOSUM62 on the default settings [59]. The sugar beet KWS2320 genotype RefBeet-1.2 proteome has been mined employing BLOSUM62 under their default settings [71]. The analyses have permitted the identification of COG genes and alternate splice variants. *G*. *max* transcriptomic data used to determine expression in leaf, nodule, pod, root, root hair, seed, shoot apical meristem (SAM) and stem has been analyzed using Phytomine in Phytozome using default parameters [49]. COG protein motifs were determined using MOTIF (MOTIF: Searching Protein Sequence Motifs (genome.jp) under default settings. Details of PFam (PF) families can be determined at https://pfam.xfam.org/ [77].

### Determination of COG complex gene expression occurring during the resistant reaction in *G*. *max*

The identification and selection of the *G*. *max* COG genes that have been used in the functional transgenic studies has occurred by using the gene expression data of Matsye et al. (2011) [28]. The procedure is summarized here for clarity. Matsye et al. (2011) have performed microarray analyses that have employed the GeneChip Soybean Genome Array (Affymetrix) [28]. In those studies, Matsye et al. (2011) [28] have infected two different *G*. *max* genotypes that are each capable of undergoing either a susceptible or resistant reaction with those reactions dependent on the *H*. *glycines* genotype used in the infections. Infection of *G*. *max*_[Peking/PI 548402]_ and *G*. *max*_[PI 88788]_ with *H*. *glycines*_[race 14/HG-type 1.3.6.7/TN8]_ leads to a susceptible reaction. In contrast, infection of *G*. *max*_[Peking/PI 548402]_ and *G*. *max*_[PI 88788]_ with *H*. *glycines*_[NL1-Rhg/HG-type 7/race 3]_ leads to a resistant reaction. The pericycle (control) cells collected at 0 days post infection (dpi) and *H*. *glycines*-parasitized syncytia undergoing the process of resistance have been collected at 3 and 6 dpi using laser microdissection (LM) [28]. These time points have been selected for specific reasons. The syncytia develop from pericycle and surrounding cells (0 dpi). Syncytia collected at an earlier stage of parasitism (3 dpi) during susceptible or resistant reactions at 3 dpi appear similar cytologically. The similarities include hypertrophy, an increase in endoplasmic reticulum (ER) and ribosome content, an enlargement of nuclei and the development of dense cytoplasm. Consequently, a 6 dpi time point is selected that functions in better differentiating between a susceptible and resistant reaction. By 6 dpi, syncytia undergoing a susceptible reaction exhibit hypertrophy of nuclei and nucleoli, have a reduction and dissolution of the vacuole, experience a proliferation of cytoplasmic organelles and exhibit an increase in cell expansion by incorporating adjacent cells. Conversely, the resistant reaction cytology is genotype-specific. The 6 dpi *G*. *max*_[Peking/PI 548402]_ resistant reaction is characterized by cells having cell wall appositions (CWAs). CWAs are structures that develop through actin polarization and vesicle-mediated delivery of cargo aggregate cytoplasmic components. Also, the 6 dpi *G*. *max*_[Peking/PI 548402]_ resistant reaction includes the production of a necrotic layer of cells that surrounds the syncytium. The cells undergoing the resistant reaction also accumulate ER, leading to the blockage of *H*. *glycines* development at the parasitic J2 stage. The *G*. *max*_[PI 88788]_ resistant reaction also has an accumulation of ER, but differs from *G*. *max*_[Peking/PI 548402]_ by lacking cell wall appositions and lacking a necrotic layer of cells that surrounds the syncytium during the resistant reaction. The *G*. *max*_[PI 88788]_ resistant reaction, however, leads to blockage of *H*. *glycines* development at J3-J4 stage [23, 24].

The cDNA probes that have been used in the Affymetrix GeneChip Soybean Genome Array (arrays) microarray hybridizations are made from the 0, 3 and 6 dpi RNA samples. The arrays are composed, in part, of 37,744 *G*. *max* probe sets. The probe sets cover 35,611 transcripts. The microarray experiments have been run in triplicate for each *G*. *max* genotype and time point under study. Consequently, the experimental process leads to the production of 6 total arrays for each time point (*G*. *max*_[Peking/PI 548402]_: arrays 1–3; *G*. *max*_[PI 88788]_: arrays 1–3). The detection call methodology (DCM) that has been used in the analysis has been implemented in Bioconductor®. The Bioconductor implementation of the standard Affymetrix® microarray DCM analysis consists of four steps. The four steps include (1) saturated probe removal, (2) discrimination score calculation, (3) Wilcoxon’s rank test p-value calculation, and (4) detection call assignment. The quantitative procedure determines whether the expression of a gene is provably different from zero (present [P]), exhibits uncertain measurement (marginal [G]), or is not provably different from zero (absent [A]). Here, a COG gene is considered measured [M] when the probe signal is detectable above threshold (p < 0.05) on all 6 arrays for a given time point. In contrast, the expression of a COG gene is considered not measured (NM) if probe signal is not detected at a statistically significant level (p ≥ 0.05) on any one of the 6 arrays using the Mann–Whitney–Wilcoxon (MWW) Rank-Sum Test which is a nonparametric test of the null hypothesis not requiring the assumption of normal distributions [78]. In some cases, there are genes that have no probe set fabricated onto the microarray. Consequently, gene expression is not determined and is not applicable (n/a). For the microarray analysis that has been performed by Matsye et al. (2011) [28], the Affymetrix annotations are mapped to the original *G*. *max* genome release Wm82.a1.v1.1 (2010). This annotation had to be used at that time (2011) because just that annotation had been available. These older annotations have undergone a comparison here to update the accessions to the more recent *Glycine max* Wm82.a2.v1 (2015) genome assembly and annotation.

### COG gene expression in COG overexpressing and RNAi transgenic roots

COG complex gene expression for the targeted COG gene and the remaining COG complex genes were examined by RT-qPCR using RNA isolated in Lawaju et al. (2020) [17]. The RNA isolation procedure is presented here for clarity. The RNA acquiring procedure involved isolation of *G*. *max* root mRNA used the UltraClean®Plant RNA Isolation Kit according to the manufacturer’s instructions (Mo Bio Laboratories®, Inc.) [17]. DNase I (Invitrogen®) has been used to remove genomic DNA. The cDNA synthesis from mRNA used the SuperScript®FirstStrand Synthesis System for RT-PCR (Invitrogen) according to the manufacturer’s instructions. The cDNA synthesis reaction employed the oligo d(T) primer according to the manufacturer’s instructions (Invitrogen). Genomic DNA contamination has been assessed using a β-conglycinin primer pair that amplifies DNA across an intron [79]. The PCR reaction yields different sized amplicons based on intron presence or absence [79]. The COG genes examined in Lawaju et al. (2020) [17] are presented here for clarity. In those analyses, Lawaju et al. (2020) [17] isolated RNA from roots individually undergoing overexpression or RNAi for each of the 16 COG *G*. *max* complex genes in biological triplicate, including COG1-1 (Glyma.10G201900), COG1-2 (Glyma.20G188500), COG2-1 (Glyma.17G129100), COG2-2 (Glyma.05G047300), COG3-1 (Glyma.13G114900), COG3-2(Glyma.17G045100), COG4-1 (Glyma.19G260100), COG4-2 (Glyma.03G261100), COG5-1 (Glyma.14G029500), COG5-2 (Glyma.02G286300), COG6-1 (Glyma.01G154500), COG6-2 (Glyma.11G090100), COG7-1 (Glyma.09G224000), COG7-2 (Glyma.12G013000), COG8-1 (Glyma.16G120600) and COG 8–2 (Glyma.02G043400). From these Lawaju et al. (2020) [17] analyses, COG1-2, COG2-2, COG3-1, COG4-2, COG5-1, COG6-1, COG7-2 and COG8-1 expression has been analyzed further here by RT-qPCR because they were the paralogs that functioned in defense. The RT-qPCR procedure is presented in the next section. RT-qPCR primers are provided (**S1 Table**).

### RT-qPCR

Confirmation of COG gene expression has been accomplished by RT-qPCR according to Lawaju et al. (2020) [17]. RT-qPCR involved RNA isolated in 2 different prior analyses [17, 32]. Firstly, RNA has been used from COG overexpression and RNAi roots and controls in experiments that demonstrated specific COG genes functioned in defense in the *G*. *max*-*H*. *glycines* pathosystem [17]. The specific COG overexpressing and RNAi roots were COG1-2, COG2-2, COG3-1, COG4-2, COG5-1, COG6-1, COG7-2 and COG8-1, along with their respective controls. Secondly, the confirmation of COG gene expression has been done on the same RNA used previously in RNA seq analyses of MAPK overexpressing roots and controls because MAPKs are important genes functioning in defense signaling processes that lead to altered transcription in the *G*. *max*-*H*. *glycines* pathosystem [32, 51]. These MAPK overexpressing roots include MAPK2 (Glyma.06G029700), MAPK3-1 (Glyma.U021800), MAPK 3–2 (Glyma.12G073000), MAPK 4–1 (Glyma.07G066800), MAPK 5–3 (Glyma.08G017400), MAPK6-2 (Glyma.07G206200), MAPK 13–1 (Glyma.12G073700), MAPK16-4 (Glyma.07G255400) and MAPK20-2 (Glyma.14G028100), in comparison to their control. Taqman 6-Carboxyfluorescein (6-FAM) labeled probes and Black Hole Quencher (BHQ1) (MWG Operon) have been used in the analysis (**S1 Table**) according to the manufacturer’s instructions. A ribosomal S21 (RPS21) protein coding gene (Glyma.15G147700) has been used as the control in the RT-qPCR experiments (**S1 Table**). The 2^-ΔΔ*C*^_T_ method of Livak and Schmittgen (2002) has been used to determine the relative change in gene expression caused by the genetic MAPK-OE engineering event as compared to the control [80]. The same approach has been employed for the RNA isolated from the COG-OE and COG-RNAi roots as compared to their respective pRAP15 and pRAP17 controls. A Student’s *t*-test has been used to calculate the p-values for the replicated RT-qPCR reactions [81]. Experiments and statistical analyses have been performed from 3 independent biological replicates [32].

### COG gene expression in MAPK overexpressing transgenic roots

RNA sequencing (RNA seq) data is available as BioProject ID PRJNA664992, Submission ID: SUB8182387 [47, 51]. Single replicate generation of RNA seq data is derived from 9 defense MAPK overexpressing roots whose gene accessions have been presented in the previous section [32]. These MAPK overexpression roots include MAPK2, MAPK3-1, MAPK 3–2, MAPK 4–1, MAPK 5–3, MAPK6-2, MAPK 13–1, MAPK16-4 and MAPK20-2 and the appropriate pRAP15 controls. The data is shown as normalized log_2_(fold change) with a p-value cutoff of < 0.05.

## Results

### Identification of *G*. *max* COG complex gene expression in *H*. *glycines*-parasitized root cells

**Fig 1:**
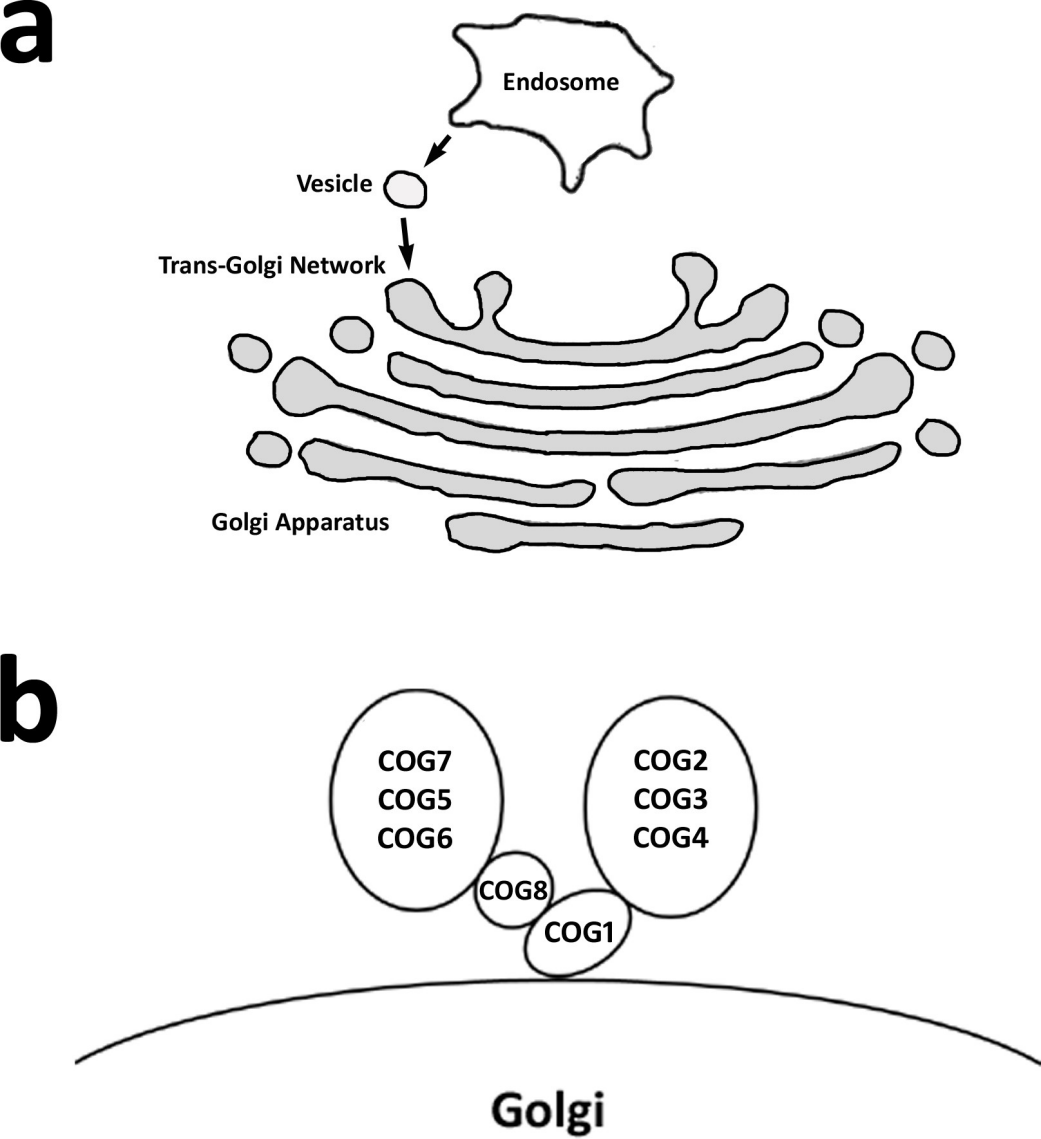
The COG complex. **a**. Vesicle trafficking occurring between the endosome and *trans*-Golgi, facilitated by the COG complex. **b**. Components of the COG complex.

The COG complex is composed of 8 proteins that regulate endosome-to-*trans* Golgi network (TGN) retrograde transport (**Fig 1**). The purpose of the first analysis presented here is to determine whether the *G*. *max* COG complex genes that have been shown to function during the resistant reaction to *H*. *glycines* parasitism are expressed within the parasitized cells undergoing a defense response [17]. This objective is relevant since the α-SNAP (*rhg1*) binding protein syntaxin 31 (SYP38) functions in defense in the *G*. *max*-*H*. *glycines* pathosystem and is known to bind COG4 and COG6 [30, 45]. To facilitate the analysis presented here, protein sequences of the eight COG complex subunits have been identified in *A*. *thaliana* and used to query the *G*. *max* proteome employing protein BLAST analyses [17]. To compliment the analysis of Lawaju et al. (2020) [17], *G*. *max* COG gene paralog accessions have been used to query a database linked to the accompanying Affymetrix microarray probe set. The results of those analyses are the identification that one *G*. *max* COG paralog for each of the 8 different COG gene families is expressed in at least one studied time point (0, 3, 6 dpi) samples relating to *H*. *glycines*-parasitized root cells undergoing a defense response in two different *G*. *max* genotypes that are capable of a defense response (**Tables 1 and S2**). Consequently, syncytium gene expression could be determined for 13 of the 16 *G*. *max* COG genes (**Tables 1 and S2**). From these analyses, COG1-2, COG2-2, COG4-2, COG5-1, COG6-1 and COG7-2 exhibit expression in analyzed RNA samples that have been obtained from at least one of the studied time point samples occurring during the resistant reaction. COG1-1, COG2-1, COG3-2, COG4-1, COG6-2, COG7-1 and COG8-2 are not observed to be expressed at the 0, 3 or 6 dpi time point samples that have been analyzed. In contrast, COG3-1, COG5-2 and COG8-1 lack probe sets on the Affymetrix microarray so gene expression could not be determined under the analysis procedures. Therefore, it is possible that COG3-1, COG5-2 and COG8-1 exhibit syncytium expression. The results largely corroborate the functional studies presented by Lawaju et al. (2020) [17] already showing that at least one COG gene family paralog functions in the defense response. Notably, COG7-2-b (Glyma.12G013000.2) is an alternative splice variant of the primary transcript COG7-2-a (Glyma.12G013000.1).

**Table 1:** *G*. *max* COG syncytium gene expression summary.

			Time point (dpi)		
Gene	Accession (Wm82.a2.v1)	Affymetrix probe set	0	3	6
COG1-1	Glyma.10G201900.2	GmaAffx.80549.2.S1_at	NM	NM	NM
*COG1-2	Glyma.20G188500.1	Gma.8255.1.S1_at	NM	NM	M
COG2-1	Glyma.17G129100.1	GmaAffx.87598.1.S1_at	NM	NM	NM
*COG2-2	Glyma.05G047300.1	Gma.7667.1.S1_a_at	M	M	M
*COG3-1	Glyma.13G114900.1	none	n/a	n/a	n/a
COG3-2	Glyma.17G045100.1	Gma.16836.1.A1_at	NM	NM	NM
COG4-1	Glyma.19G260100.1	GmaAffx.18638.1.S1_at	NM	NM	NM
*COG4-2	Glyma.03G261100.1	Gma.1626.1.S1_at	M	M	M
*COG5-1	Glyma.14G029500.1	GmaAffx.16900.1.S1_at	NM	M	M
COG5-2	Glyma.02G286300.1	none	n/a	n/a	n/a
*COG6-1	Glyma.01G154500.1	GmaAffx.51551.1.S1_at	NM	NM	M
COG6-2	Glyma.11G090100.1	GmaAffx.58162.1.S1_at	NM	NM	NM
COG7-1	Glyma.09G224000.1	GmaAffx.62631.1.S1_at	NM	NM	NM
*COG7-2	Glyma.12G013000.2	GmaAffx.61157.1.S1_at	NM	NM	M
*COG8-1	Glyma.16G120600.1	none	n/a	n/a	n/a
COG8-2	Glyma.02G043400.1	GmaAffx.47025.1.S1_at	NM	NM	NM

Each experiment has been replicated. There have been three independent biological replicates for each *G*. *max* genotype, time point and controls. The replicated experiments have happened on three different microarrays (arrays) per *G*. *max H*. *glycines*-resistant genotypes (genotype 1 is *G*. *max*_[Peking/PI 548402]_ and genotype 2 is *G*. *max*_[PI 88788]_). Red, measured expression (M); blue, not measured expression (NM); n/a, not applicable (gray) because no probe set existed on the microarray (Klink et al. 2010). The analysis of the results has occurred using data derived from the three independent replicates, analyzed by (p < 0.05, MWW) (Mann and Whitney, 1947). (*) indicates genes that function in the defense response. The raw data is provided (**S2 Table**).

### COG complex gene family structure present in other agriculturally significant plant species

COG complex genes are important to the defense process in other plant species, making a broad understanding of the complex relevant [41]. Recent studies have presented the 10 most significant agricultural plant species [82]. In addition to *G*. *max*, these plant species include *M*. *esculenta*, *Z*. *mays*, *O*. *sativa*, *T*. *aestivum*, *H*. *vulgare*, S. *bicolor*, *B*. *rapa*, *E*. *guineensis* and *S*. *officinalis* [83]. Analyses are presented here that identify the COG gene families and their structure in these crops and others that are significant components of U.S. agriculture including *B*. *vulgaris*, *S*. *tuberosum*, *S*. *lycopersicum* and *G*. *hirsutum*. To accomplish these analyses, *A*. *thaliana* COG proteins have been queried into the proteomes of *G*. *max*, *M*. *esculenta*, *Z*. *mays*, *O*. *sativa*, *T*. *aestivum*, *H*. *vulgare*, *S*. *bicolor*, *B*. *rapa*, *S*. *lycopersicum*, *S*. *tuberosum* and *G*. *hirsutum* found at Phytozome [49]. These analyses have been complimented by querying the *A*. *thaliana* COG proteins to the proteomes of *E*. *guineensis*, *S*. *officinalis* and *B*. *vulgaris* [59, 70, 71]. The results of those analyses are the identification of the gene family structure of the COG genes in those plant species (**Tables 2 and S3–S17**). Duplication of some COG genes are observed. Notably, some COG genes are tandemly duplicated (**Table 3**). These tandemly duplicated COG genes include the *M*. *esculenta* COG4 (Manes.05G016300, Manes.05G016400), *S*. *lycopersicon* COG3 (Solyc07g017520.2.1, Solyc07g017530.2.1) and *G*. *hirsutum* (Gohir.D08G170700, Gohir.D08G170800) (**Tables 3 and S3–S17**). A larger quantity of segmental duplication is observed than tandem duplication (**Tables 3 and S3–S17**). Some conclusions can be drawn from Lawaju et al. (2020) [17] regarding the specialization of function (neofunctionalization) of some of the *G*. *max* COG genes. The Lawaju et al. (2020) analysis demonstrated that COG1-2, COG2-2, COG3-1, COG4-2, COG5-1, COG6-2, COG7-2 and COG8-1 have a defense role [17]. Therefore, with regard to neofunctionalization, COG1-2, COG2-2, COG3-1, COG4-2, COG5-1, COG6-2, COG7-2 and COG8-1 have a defense role that COG1-1, COG2-1, COG3-2, COG4-1, COG5-2, COG6-1, COG7-1 and COG8-2 appear to lack. Furthermore, COG3-1, COG4-1 and COG5-1 also have a role in root growth that appears to be lacking in for the other COG genes [17]. COG3-1 and COG4-1 overexpression decreases root mass while COG3-1 and COG4-1 RNAi increases root mass. COG5-1 overexpression and RNAi increase root mass. COG3-1 and COG5-1 also have a defense role while COG4-1 does not, possibly indicating neofunctionalization. Furthermore, COG1-2, COG7-2 and COG8-1 are induced by harpin treatment while COG1-1, COG7-1 and COG8-2 are not, indicating neofunctionalization [17]. COG4-2 and COG5-1 overexpression increases syntaxin 31 transcript abundance while COG4-2 and COG5-1 RNAi decreases syntaxin 31 transcript abundance. This coupled influence on COG4-1 and COG5-2 is not observed, indicating neofunctionalization [17].To obtain a greater understanding of potential neofunctionalization, the COG protein sequences obtained from the plant genomes have been analyzed using MOTIF in cases where clear duplication has occured. *T*. *aestivum* was not analyzed here because the protein sequences appear to be largely composed of fragments of unclear nature. Consequently, 8 proteomes have been analyzed, including *G*. *max*, *B*. *rapa*, *G*. *hirsutum*, *M*. *esculenta*, *E*. *guineensis*, *S*. *tuberosum*, *S*. *officinalis* and *Z*. *mays* (**S18 Table**). In contrast, *H*. *vulgare*, *O*. *sativa*, *S*. *bicolor*, *S*. *tuberosum* and *B*. *vulgaris* have not been analyzed since they lacked duplication of their COG genes. Based off the MOTIF-determined annotations, it appears as though some of the *G*. *max* COG paralogs have differences in their deduced protein structure which could lead to the differences in properties that have been described previously. For example, COG1-1 has vacuolar protein sorting 51 (Vps51) (PFAM: PF08700), Secretion 5 (Sec5) (PFAM: PF15469), dependent on RIC1 (Dor1) (PF04124) and KxDL (PF10241) domains while COG1-2 only has Vps51 and Dor1 domains. In contrast, COG2-1 and COG2-2 both have COG2 (PF06148), VPS51 and domain of unknown function (DUF) 3510 (DUF3510) (PF12022) domains, indicating they may have similar functions even though COG2-2 is constitutively expressed and functions in defense while COG2-1 is not [3]. Other notable observations for the *G*. *max* COG proteins sequences are the presence of a COG3-3 (Glyma.09G134300) and COG6-3 (Glyma.20G085000), apparently the products of gene truncations caused by premature stop codons. Other examples of truncated COG genes were identified in, but not limited to *B*. *rapa* COG4 (Brara.E02159) and COG5 (Brara.A01419), *S*. *lycopersicon*, COG3 (Solyc07g017530.2.1), *G*. *hirsutum* COG4 (Gohir.A06G116700), *M*. *esculentum* COG2 (Manes.14G052400, COG4(Manes.05G016300.1 and Manes.05G016400). *E*. *guineensis* COG5 (p5.00_sc00515_p0005) and COG7 (p5.00_sc00013_p0068) are truncated in length as compared to their paralogs (**S18 Table**). *T*. *aestivum* had many COG gene fragments which require further confirmation of their nature before they can be adequately assessed here and therefore have not been included in this analysis.

**Table 2:** COG paralogs in select plant species.

Plant	COG1	COG2	COG3	COG4	COG5	COG6	COG7	COG8
**thale cress** ^1^	**1 (4)**	**1**	**1 (2)**	**1 (2)**	**1**	**1**	**1**	**1**
**soybean** ^2^	**2 (3)**	**2 (5)**	**3 (5)**	**3**	**2 (4)**	**3**	**2 (4)**	**2 (3)**
**cassava** ^3^	**1 (2)**	**2**	**2 (3)**	**3**	**2**	**1**	**1**	**1**
**maize** ^4^	**2 (5)**	**2 (4)**	**1 (2)**	**2 (3)**	**1**	**1**	**1**	**2 (6)**
**rice** ^5^	**1**	**1 (2)**	**1**	**1**	**1**	**1**	**1**	**1**
**wheat** ^6^	**6 (12)**	**3 (9)**	**14 (35)**	**3 (5)**	**4 (10)**	**5 (21)**	**3 (6)**	**3 (14)**
**barley** ^7^	**1 (4)**	**1 (4)**	**1 (4)**	**1 (2)**	**1 (4)**	**1 (2)**	**1 (31)**	**1 (24)**
**sorghum** ^8^	**1**	**1 (2)**	**1**	**1**	**1**	**1**	**1**	**1**
**rape seed** ^9^	**2**	**3**	**1**	**2**	**2 (3)**	**1**	**2**	**2 (3)**
**oil palm** ^10^	**2**	**2**	**1**	**2 (3)**	**2**	**1**	**2**	**1**
**sugar cane** ^11^	**1**	**1**	**2**	**1**	**1**	0*	**1**	0*
**sugar beet** ^12^	**1**	**1**	**1**	**1**	**1**	**1**	**1**	**1**
**tomato** ^13^	**1**	**1**	**2 (3)**	**1**	**1**	**1**	**1**	**1**
**potato** ^14^	**1**	**1**	**1**	**1 (3)**	**1**	**1 (3)**	**1 (2)**	**1**
**cotton** ^15^	**4 (5)**	**2 (4)**	**4 (5)**	**4 (5)**	**2 (3)**	**3 (5)**	**2**	**2**

Genome sequencing information

(1) Arabidopsis Genome Initiative, 2000; Lamesch et al. 2012

(2) Schmutz et al. 2010

(3) Bredeson et al. 2016

(4) Schnable et al. 2009

(5) Ouyang et al. 2007

(6) International Wheat Genome Sequencing Consortium (IWGSC).

(7) Mascher et al. 2017; Beier et al. 2017

(8) McCormick et al. 2017

(9) Wang et al. 2011; Zhang et al. 2018

(10) Singh et al. 2013

(11) Garsemeur et al. 2018

(12) Dohm et al. 2014

(13) Tomato Genome Consortium, 2012

(14) Potato Genome Sequencing Consortium, 2011

(15) Zhang et al. 2015; Saski et al. 2019; Wang et al. 2019. Genome details are presented in the Materials and Methods section. Please refer to **S3–S17 Tables**.

**Table 3:** COG genes that have experienced duplication in the studied plants.

Scientific name	common name	COG1	COG2	COG3	COG4	COG5	COG6	COG7	COG8
*Glycine max*	soybean	1	1	1	1	1	1	1	1
*Arabidopsis thaliana*	thale cress	0	0	0	0	0	0	0	0
*Hordeum vulgare*	barley	0	0	0	0	0	0	0	0
*Manihot esculenta*	*cassava*	0	1	1	3	1	0	0	0
*Zea mays*	maize	1	1	0	1	0	0	0	1
*Oryza sativa*	rice	0	0	0	0	0	0	0	0
*Triticum aestivum*	wheat	1	1	1	1	1	1	1	1
*Sorghum bicolor*	sorghum	0	0	0	0	0	0	0	0
*Brassica bicolor*	rape	1	1	0	1	1	0	1	1
*Elaes guineensis*	oil palm	1	1	0	1	1	0	1	0
*Saccharum officinalis*	sugar cane	0	0	1	0	0	X	0	X
*Solanum lycopersicum*	tomato	0	0	1	0	0	0	0	0
*Solanum tuberosum*	potato	0	0	0	0	0	0	0	0
*Gossypium hirsutum*	cotton	1	1	1	1	1	3	1	1
*Beta vulgaris*	sugar beet	0	0	0	0	0	0	0	0

0, not duplicated.

1, segmental.

2, tandem.

3, 1 and 2.

X gene not identified.

### COG gene families have alternative splice variants expressed in root cells undergoing defense

**Fig 2:**
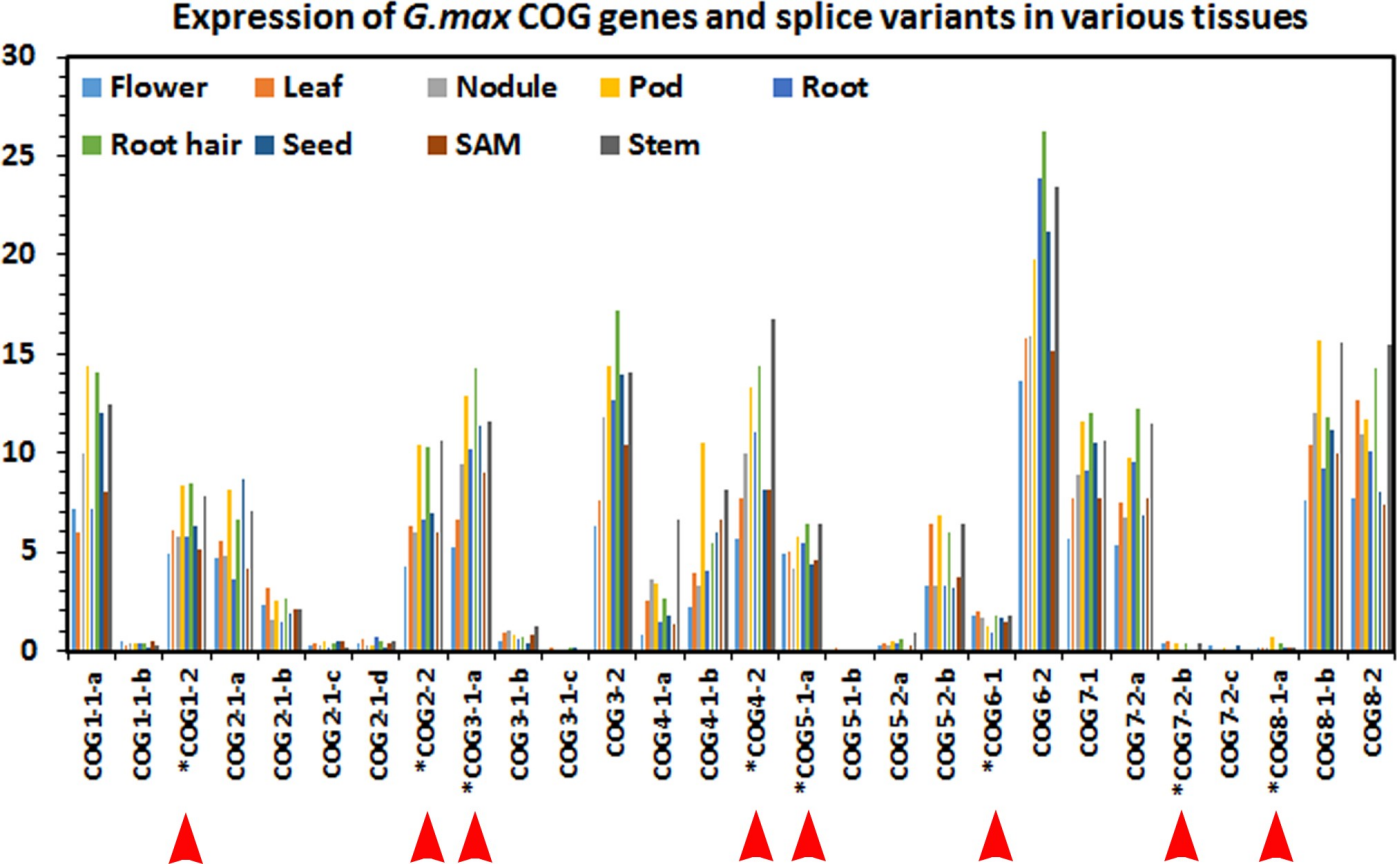
COG RNA seq expression abundance in different tissue types in *G*. *max*. * along with the red arrowhead indicates the gene functions in defense (Lawaju et al. 2020 [17]). In this image, the spice variants are labeled. For example, COG7-2-a is Glyma.12G013000.1, COG7-2-b is Glyma.12G013000.2 and COG7-2-c is Glyma.12G013000.3. COG7-2-b is Glyma.12G013000.2 is the examined splice variant that functions in defense (Lawaju et al. 2020 [17]). Gene expression data has been obtained from Phytomine in Phytozome (Libult et al. 2010; Goodstein et al. 2012 [49]; Wang et al. 2019).

The analyses presented in **Tables 2** and **S3–S17** also provide the alternative splice variants for COG genes. This facet of COG gene transcription may be relevant to the ability that plants have in defending themselves from pathogenic attack [41]. The *H*. *vulgare* defense gene *Hv*COG3 (HORVU7Hr1G062190) has 4 alternate splice variants, indicating that perhaps there are specific variants that may confer specialized functions that facilitate the defense role as shown in *G*. *max* for COG7-2-b (Glyma.12G013000.2) [17]. To examine COG complex splice variant structure, the *G*. *max* COG gene expression data available in Phytozome has been mined using Phytomine and examined for whether the RNA seq data confirms the expression of these alternate splice variants. The results show the expression of each COG splice variant in relation to 9 different tissue types, including leaf, nodule, pod, root, root hair, seed, shoot apical meristem (SAM) and stem (**Fig 2**). Each sample type, except for the root hair sample is a plant organ so a clear understanding of the individual cellular expression profiles could not be performed. Unfortunately, since these data have been obtained from a public data base (Phytozome), it was not possible to examine these same RNA samples using RT-qPCR. An examination of the *G*. *max* microarray data in relation to these splice variants show that specific splice variant data is available for some of the COG genes. The only COG gene whose alternate splice variant has been shown to function in defense is COG7-2, splice variant 2 (COG7-2-b [Glyma.12G013000.2]). However, in that analysis, each individual *G*. *max* COG gene splice variant has not been examined in functional analyses because they were beyond the scope of that analysis and the analysis presented here of [17].

In contrast, RNA seq analysis have identified that some COG genes, including specific splice variants, are not expressed in the *G*. *max* MAPK overexpression roots as compared to controls (**S19 Table**). Some of these splice variants are expressed in either some or all of the examined tissue types including COG2-1, COG3-1, COG4-1, COG5-2 and COG7-2 in seed, flower, nodules, root, SAM, root hair, leaves, pods and stems [49]. COG2-1 (Glyma.17G129100.4) is expressed in all of the sample types, but has not been observed in syncytia undergoing a defense response under the analysis procedures, nor tested for a function in the defense process [17, 49]. COG3-1 (Glyma.13G114900.3) is not expressed in nodules, roots, SAM and stems (**S19 Table**) [49]. COG3-1 expression in syncytia could not be determined due to the analysis procedures, but does function in the defense process *G*. *max* has to *H*. *glycines* [17]. COG5-2 (Glyma.02G286300.2) is expressed in all samples except in seed and is not expressed in syncytia and does not function in the *G*. *max* defense process to *H*. *glycines* (**S19 Table**) [17, 49]. The COG7-2 (Glyma.12G013000.3) is not expressed in roots or stems and has not been examined in functional experiments, testing if it functions in defense in *G*. *max* to *H*. *glycines* (**S19 Table**) [17, 49].

### COG genes exhibit co-regulated expression

**Fig 3:**
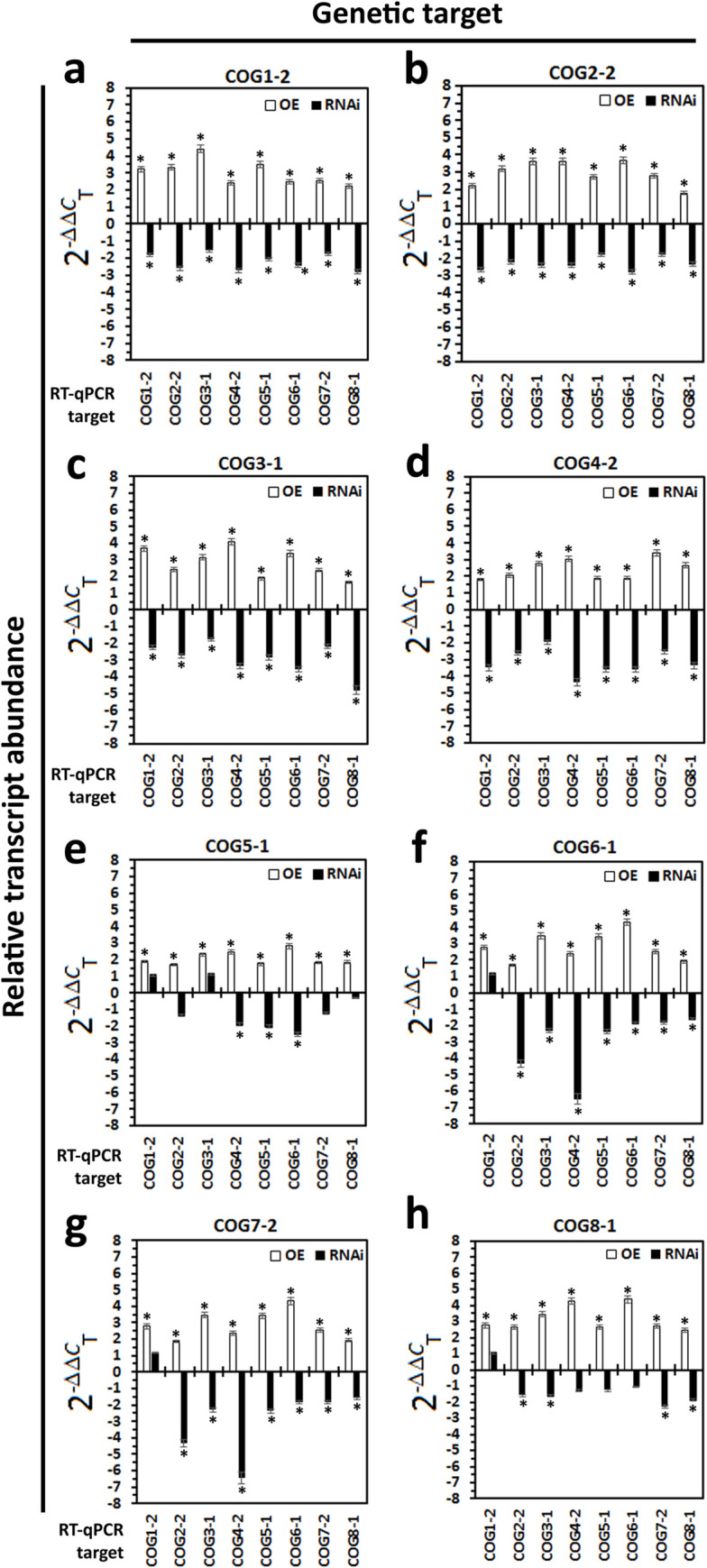
RT-qPCR analysis of COG gene expression in COG-OE and COG-RNAi transgenic roots. The COG transgenic roots are **a**. COG1-2 (Glyma.20G188500), **b**. COG2-2 (Glyma.05G047300), **c**. COG3-1 (Glyma.13G114900), **d**. COG4-2 (Glyma.03G261100), **e**. COG5-1 (Glyma.14G029500), **f**. COG6-1 (Glyma.01G154500), **g**. COG7-2 (Glyma.12G013000) and **h**. COG8-1 (Glyma.16G120600) in comparison to the appropriate pRAP15 and pRAP17 controls. The control gene is RPS21 (Glyma.15G147700), Lawaju et al. 2020 [17]. The 2^-ΔΔ*C*^_T_ method has been used to determine the relative change in COG gene expression (the RT-qPCR target) caused by the COG-OE or COG-RNAi genetic engineering event as compared to the control (Livak and Schmittgen 2002). *Statistically significant, Student’s t-test p < 0.05.

Experiments show that co-regulated gene expression exists between SNARE genes in the *G*. *max*-*H*. *glycines* pathosystem [30, 38]. Roots undergoing COG complex gene overexpression or RNAi have had their RNA isolated from whole transgenic roots, unlike the prior experiments examining gene expression of specific cells (pericycle and syncytia) collected by LM [17]. The RNA has been used in a series of RT-qPCR experiments examining the level of expression for each COG complex component shown to function in the defense process (**Fig 2**). In some cases, co-regulated gene expression is observed whereby the overexpression/RNAi of one COG gene influences the relative transcript abundance of another COG gene while the affected COG gene, when engineered for overexpression or RNAi affects the relative transcript abundance of the other COG gene in the examined pair in the same manner. Similar results have been obtained in the *G*. *max*-*H*. *glycines* pathosystem for other genes functioning in vesicle transport and specific MAPKs [30, 32, 38]. In the experiments presented here, roots engineered to undergo overexpression or RNAi of the COG genes have already been produced and had their RNA isolated [17]. Those samples have been used to examine by RT-qPCR the relative transcript abundance of the COG gene targeted for transgenic overexpression or RNAi, along with the other COG genes that function in the defense response (**Fig 3**).

### COG gene expression in defense MAPK overexpressing roots

**Fig 4:**
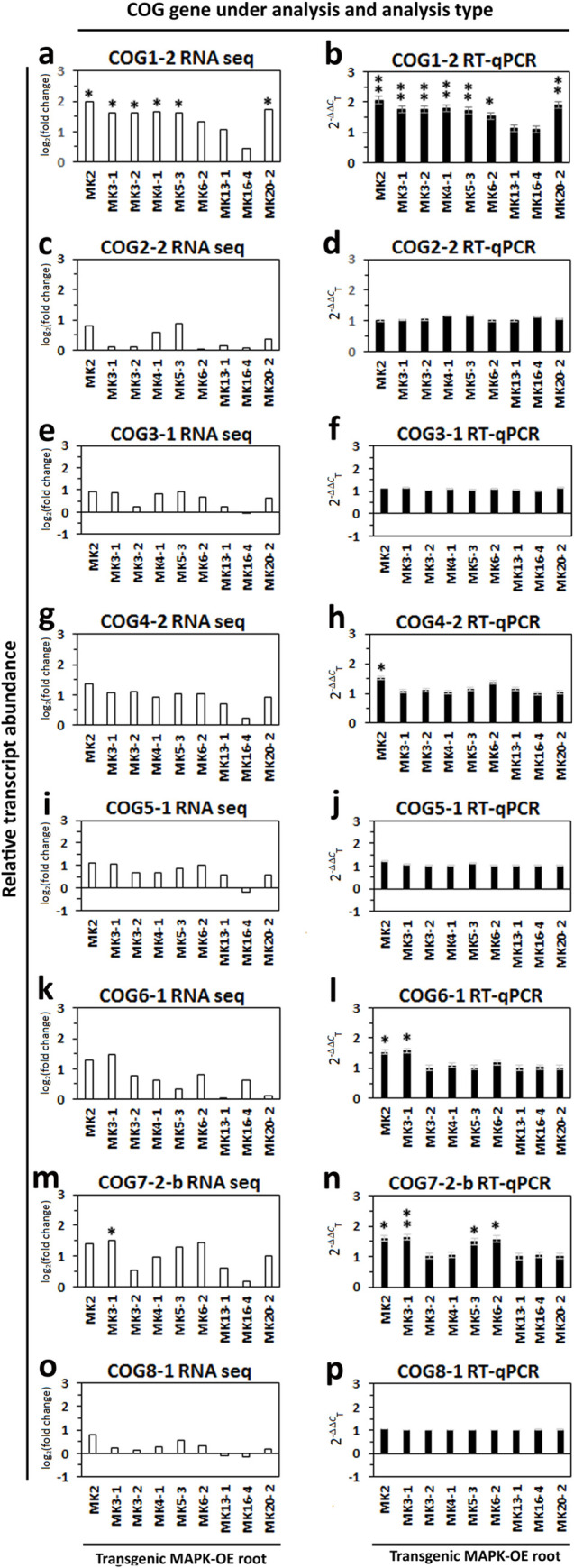
Relative transcript abundance of COG genes in MAPK-overexpressing roots. **a**. COG1-1 analyzed by RNA seq, **b**. COG1-2 RNA analyzed by RT-qPCR, **c**. COG2-2 analyzed by RNA seq, **d**. COG2-2 analyzed by RT-qPCR, **e**. COG3-1 analyzed by RNA seq, **f**. COG3-1 analyzed by RT-qPCR, **g**. COG4-2 analyzed by RNA seq. **h**. COG4-2 analyzed by RT-qPCR. **i**. COG5-1 analyzed by RNA seq, **j**. COG5-1 analyzed by RT-qPCR, **k**. COG6-1 analyzed by RNA seq, **l**. COG6-1 analyzed by RT-qPCR, **m**. COG7-2-b analyzed by RNA seq, **n**. COG7-2-b analyzed by RT-qPCR, **o**. COG8-1 analyzed by RNA seq, **p**. COG8-2 analyzed by RT-qPCR. Single replicate RNA seq analyses have been performed of RNA isolated from MAPK overexpressing roots. These results have been confirmed by RT-qPCR. The MAPK overexpressing roots include MAPK2 (Glyma.06G029700), MAPK3-1 (Glyma.U021800), MAPK 3–2 (Glyma.12G073000), MAPK 4–1 (Glyma.07G066800), MAPK 5–3 (Glyma.08G017400), MAPK6-2 (Glyma.07G206200), MAPK 13–1 (Glyma.12G073700), MAPK16-4 (Glyma.07G255400) and MAPK20-2 (Glyma.14G028100) and the appropriate pRAP15 control. The RNA seq data is shown as normalized log_2_(fold change) with a p-value cutoff of < 0.05. The RT-qPCR data is shown after employing the 2^-ΔΔ*C*^_T_ method of Livak and Schmittgen (2002) to determine the relative change in COG gene expression caused by the MAPK-OE genetic engineering event as compared to the control. *Statistically significant and meeting the 1.5 fold induced criteria, Student’s t-test p < 0.05. **Statistically significant and meeting the 1.5 fold induced criteria in RNA seq and RT-qPCR analyses.

Analyses show that 9 *G*. *max* MAPKs out of the 32 occurring in the *G*. *max* genome function in the defense response to *H*. *glycines* [32]. To obtain an understanding of the potential regulation of COG gene expression relating to defense in the *G*. *max*-*H*. *glycines* pathosystem, RNA-seq data has been generated from RNA isolated from whole roots overexpressing the defense MAPKs and their control. The results are presented (**Fig 4**). In certain cases, MAPK overexpression leads to an increase in relative transcript abundance of at least 1.5 fold, p < 0.05, of certain COG genes when examining the RNA seq data. Induced COG1-2 gene expression is observed in MAPK2-OE (1.99 fold), MAPK3-1-OE (1.61 fold), MAPK3-2-OE (1.61 fold), MAPK4-1-OE (1.67 fold), MAPK5-3-OE (1.63 fold) and MAPK20-2-OE (1.74 fold) roots as compared to their controls. The relative transcript abundance of COG1-2 in the MAPK6-2-OE (1.34 fold), MAPK13-3-OE (1.07 fold) and MAPK16-4-OE (0.43 fold) roots are statistically significant (p < 0.05), but did not meet the criteria of the level of induced expression (1.5 fold or greater) as compared to their controls. Similar results are observed in the RT-qPCR experiments. In the RT-qPCR analyses, increased COG1-2 transcript abundance is observed in the MAPK2-OE (2.07 fold), MAPK3-1-OE (1.77 fold), MAPK3-2-OE (1.75 fold), MAPK4-1-OE (1.81 fold), MAPK5-3-OE (1.71 fold), MAPK6-2-OE (1.54 fold) and MAPK20-2-OE (1.91 fold) roots as compared to their controls. The relative transcript abundance of COG1-2 in the MAPK13-1-OE (1.13 fold) and MAPK16-4-OE (1.11 fold) roots did not meet either of the induced expression criteria as compared to their controls.

RNA seq analyses identify induced COG4-2 gene expression to be statistically significant (p < 0.05) in the MAPK4-1-OE roots, but did not meet the criteria (≥ 1.5 fold) for induced expression (1.35 fold) as compared to their controls. However, the RT-qPCR analyses determine change in COG4-2 gene expression is statistically significant and meeting the ≥ 1.5 fold criteria in the MAPK4-1-OE roots (1.51 fold) as compared to controls.

RNA seq analyses identify that induced COG6-1 gene expression is statistically significant (p < 0.05) in the MAPK2-OE and MAPK3-1-OE roots as compared to their controls, but did not meet the criteria (≥ 1.5 fold) for induced expression (1.29 fold and 1.48 fold, respectively). However, the RT-qPCR analyses determine COG4-2 gene expression is induced at a statistically significant level and meeting the ≥ 1.5 fold criteria in the MAPK2-OE (1.53 fold) and MAPK3-1-OE (1.58 fold) roots as compared to their controls.

RNA seq analyses identify induced COG7-2-b gene expression is statistically significant (p < 0.05) only in the MAPK3-1-OE roots (1.5 fold) as compared to their controls. In contrast, RNA seq analyses identify induced COG7-2-b gene expression is statistically significant (p < 0.05) in the MAPK2-OE, MAPK5-3-OE and MAPK6-2-OE roots, but did not meet the criteria (≥ 1.5 fold) for induced expression (1.42, 1.29, 1.42 fold, respectively) as compared to their controls. However, the RT-qPCR analyses determine COG7-2-b gene expression is induced at a statistically significant level and meeting the ≥ 1.5 fold criteria in the MAPK2-OE (1.61 fold), MAPK3-1-OE (1.63 fold), MAPK5-3-OE (1.53 fold) and MAPK6-2-OE (1.59 fold) roots as compared to their controls.

## Discussion

The goal of the analysis presented here is to analyze data to complement previous studies involving functional transgenic experiments performed in *G*. *max*, examining the role that its COG genes have during its defense response to *H*. *glycines* parasitism [17]. That goal, obtained in the analysis presented here, allows for an understanding of the COG genes more broadly across different plant species by revealing the expression they have prior to and during the *G*. *max* defense process [17, 41]. The experiments of Lawaju et al. (2020) [17] identify the existence of two COG complex paralogs for each of the 8 *G*. *max* COG genes. The experiments of Lawaju et al. (2020) [17] then demonstrated that only one of the two paralogs of each COG gene family functions in the defense process that *G*. *max* has toward *H*. *glycines* parasitism. However, in all, one COG gene of each of the 8 COG complex gene families functions in defense [17]. The role that the COG complex, as a vesicle transport component, performs in homeostasis may have broad implications regarding plant responses to pathogens in general, newly emerging pathogens and to climate change, making this study important [17, 82–85]. Because of these roles, analyses done to identify COG genes and their potential splice variants in other important crop species and obtaining an understanding of their regulated expression have been done here.

### The relationship between COG complex genes and defense

The presented results are important from the standpoint that the syntaxin 31 homolog of *S*. *cerevisiae*, Sed5p, binds to Sec17p, COG4 and COG6 [43–46]. Notably, the *G*. *max* Sec17p homolog, α-SNAP-5, is stated as being the major *H*. *glycines* resistance gene *rhg1* although the locus is complex in nature [28–30, 38, 39]. Therefore, the results presented here clearly link the vesicle transport system and membrane fusion apparatus to the defense process that *G*. *max* has toward *H*. *glycines* parasitism [40]. Understanding where, how and why various genes are expressed during the defense process and the ordering of the expression of those genes will provide needed insight into the cellular processes that underlie resistance and the functionality of the COG complex not only in *G*. *max*, but broadly in different plant species [86, 87].

### Expression of the COG complex genes during the resistant reaction

A comparative analysis of the *G*. *max* COG genome accessions identified here is made to accessions accompanying previously reported gene expression patterns occurring within the root cells relating to its resistant reaction (syncytium) to *H*. *glycines*. Analyses presented here determine that 13 of the 16 *G*. *max* COG complex genes (81.25%) had Affymetrix probe sets on the GeneChip Soybean Genome Array, including at least one COG complex component from each of its 8 gene families [88]. The analyses presented here then identify COG complex gene expression occurring within the syncytium undergoing a resistant reaction. The analyses show that COG2-2, COG4-2 and COG5-1 have measurable expression within the pericycle and surrounding cells (control) at 0 dpi prior to *H*. *glycines* infestation of the soil. However, by 6 dpi which would be at a time point occurring as the resistant reaction is concluding, measurable COG gene expression within the syncytium is observed for COG1-2, COG2-2, COG4-2, COG5-1, COG6-1 and COG7-2-b. Consequently, expression is detected within the cells undergoing a resistant reaction for members of 6 of the 8 COG gene families. In contrast, the detection of expression for *G*. *max* COG3-1 and COG8-1 paralogs could not be made under the analysis procedures due to the lack of probe sets on the microarray, remaining avenues of research for future study.

The COG complex gene expression identified here to be occurring within certain cell types indicates that these genes may serve a basic function in the cell biology of *G*. *max*. This observation is in agreement with the original observations made in *S*. *cerevisiae* for Sec35p (COG2), Sec38p (COG4) and Cod4p (COG5) [3, 11, 12, 89]. In contrast, the *G*. *max* COG1-2 (Sec36p/Cod3p), COG6-1 (Sec37p/Cod2p) and COG7—b (Cod5p) exhibit measurable amounts of gene expression only at 6 dpi. Therefore, it appears that unlike the other COG complex genes, COG1-2, COG6-1 and COG7-2-b may exhibit a level of gene regulation that is related specifically to the development of the resistant reaction in *G*. *max* in the syncytium. Studies performed on *H*. *vulgare* demonstrate *Hv*COG3 functions during the resistant reaction to fungal penetration into the host cell [41]. Consequently, it is not without precedent that multi-subunit structures requiring all of its components are important for the integrity of the structure. For example, the exocyst functions upstream of membrane fusion at the tethering stage of vesicle transport in relation to SNARE which acts downstream at docking stage [90, 91]. The exocyst requires all 8 of its component parts for the functionality of the structure [92]. Not surprising, the elimination of even one component leads to the loss of function of the structure [92]. Consistent with this observation, the expression of each component of the *G*. *max* exocyst, like its COG complex, is important to its defense process to *H*. *glycines* parasitism [17, 93].

In *A*. *thaliana*, its COG7 ortholog, *embryo yellow* (*EYE*) gene functions in the maintenance of the meristem, indicating a specialized role in its cellular biology and metabolism [16]. The *eye* mutants are bushy, have SAMs with aberrant organization and have an altered composition of their cell walls [16]. This is an important observation because in *G*. *max* the secreted, hemicellulose-modifying gene xyloglucan endotransglycosylase/hydrolase (XTH), XTH43, is one of the most highly expressed genes in the syncytium undergoing a defense response [28]. XTH43 also has a significant role in the resistant reaction to *H*. *glycines* [30]. XTH43 increases xyloglucan (XyG) content, shortens XyG chains and makes more of those shorter chains while it can also be expressed in other plants (i.e. *G*. *hirsutum*) to generate a defense response to the root knot nematode *Meloidogyne incognita* where one does not exist [20, 40]. XTH is targeted to the Golgi apparatus prior to its secretion into the apoplast where it functions in cell wall modification [94–96]. The Golgi apparatus, thus, serves prominently in processes involving cell wall modification, requiring the import of enzymes and glycoproteins from the ER to the Golgi via transition vesicles [97, 98]. However, the synthesis of XyG and modification of XyG, itself, occurs in the Golgi apparatus, first in the cisternae then moving to the medial- and trans- Golgi as XyG matures [99, 100]. Transport of the matrix polysaccharides and enzymes to the cell membrane then occurs through secretory vesicles [101]. In the experiments presented here 7 of the targeted COG complex genes are shown to have probe sets fabricated onto the microarray, but those genes did not exhibit measurable amounts of expression (i.e., COG1-1, COG2-1, COG3-2, COG4-1, COG6-2, COG7-1 and COG8-2). The remaining 3 *G*. *max* COG complex genes (COG3-1, COG5-2 and COG8-1) did not have corresponding probe sets fabricated onto the array, complicating an understanding of the relationship of these COG complex genes to the resistant reaction under study here. Since the *Hv*COG3 has been shown to function in the resistant reaction in wheat, it was going to be important to the understanding of the complex to analyze the entire *G*. *max* COG complex in transgenic functional analyses to obtain a clear understanding of the structure in relation to the resistant reaction to *H*. *glycines* parasitism as shown in Lawaju et al. (2020) [17].

### The complexity of the COG complex gene families

The COG complex is an integrated structure made challenging to understand because of the intricate nature of the plant genome, with all plant genomes believed to have undergone polyploidization events [102]. These events are then followed by rearrangement and/or reduction that have various effects on growth and development [103]. For example, *A*. *thaliana* has experienced 3 genome duplication events referred to as paleopolyploidy [104, 105]. During its evolutionary history there was an initial paleohexaploidy event that occurred in the asterales and rosids, followed by a paleotetraploidy event that was limited to the Brassicales [104, 105]. Subsequent genome rearrangement and reduction then occurred [104, 105]. While the genome of the *A*. *thaliana* ancestors underwent these duplication events, it is functionally diploid (2n = 10) [52]. The diploid nature of the *A*. *thaliana* genome is reflected in a single COG gene existing for each of the 8 COG members [17, 52]. *A*. *thaliana* COG complex protein sequences have been used to mine the available genomes of agriculturally important crops on a world-wide scale and then some more specific to the U.S. The proteome mining of several plant genomes with the *A*. *thaliana* COG sequences is consistent with the diploid nature of other species presented here. *O*. sativa (2*n* = 24), *H*. *vulgare* (2n = 14), *S*. *tuberosum* (double monoploid Phureja DM1-3 516 R44, 2n = 24), *S*. *bicolor* (2n = 20) and the *B*. *vulgaris* (2n = 18) KWS2320 reference genomes are diploid [64, 71, 106, 107]. However, *S*. *tuberosum* is typically tetraploid (2n = 4x = 48) and *B*. *vulgaris* can also be triploid [64, 71]. Results of the proteomic analyses are consistent with the diploid nature of *O*. *sativa*, *H*. *vulgare*, *B*. *vulgaris* and *S*. *tuberosum* in that they lack duplication of any of their COG genes. Some of the plants that have been studied here are also diploids, but have a limited amount of COG gene duplication. For example, *S*. *lycopersicon* (2n = 24) has a duplication limited only to COG3 with the rest of the COG genes not existing as duplicated gene families [108, 109]. The duplicated SlCOG3 is not the product of localized gene amplification, a process shown to be important in generating plant defense capabilities [36]. *E*. *guineensis* is diploid (2n = 32), but has duplications of COG1, 2, 4, 5 and 7. It is possible that further analysis of the *E*. *guineensis* genome may reveal its genetic structure is more complicated with various levels of polyploidy since its current coverage is at 79% [72]. Evidence has been presented that *E*. *guineensis* has experienced 2 polyploidization events, one that is lineage specific and one that is more evolutionarily broad, found in commelinid plants [61]. The remainder of the plants under study, *G*. *max*, *Z*. *mays*, *T*. *aestivum*, *B*. *rapa*, *S*. *officinalis* and *G*. *hirsutum* are polyploid. *G*. *max*, (2n = 40) is an allotetraploid with each of its COG genes being at least duplicated [62]. *B*. *rapa*, as a member of the Brassicaceae, has shared the same evolutionary history as *A*. *thaliana*, but with the addition of a whole-genome triplication (WGT) event that is believed to have occurred, resulting in a mesohexaploid [68, 110, 111]. The diploid *B*. *rapa* genome (2n = 2x = 20) has duplicated COG1, 2, 4, 5 and 8 [112]. *Z*. *mays* (2n = 20) is a replicated diploid having undergone a whole genome duplication as a paleopolyploid with a subsequent duplication that differentiates it from *S*. *bicolor* [113]. *Z*. *mays*, consequently unlike *S*. *bicolor*, has 2 copies of COG1, 2, 4 and 8 with the rest of the gene families having a single copy. *T*. *aestivum*, a hexaploid having 2 copies of its AB and D genomes (2n = 6x = 42), has duplicated COG genes for each of its family members in multiples of 3 except for COG5 (4 copies) and COG6 (5 copies) [114]. The confirmation and annotation of these genes appears to require more work since some of the duplicated gene sequences appear to be fragments that are not the result of premature stop codons. The *S*. *officinalis* genome is a polyploid (2n = 8x = 80) with the generation of an accurate genome has been hampered by its polyploid nature [115]. Currently, COG3 is understood as being duplicated. COG1, 2, 4, 5 and 7 are not duplicated. Homologs of COG6 and COG8 could not be identified even in BLASTP searches of the available sequences deposited in Genbank. The *G*. *hirsutum*, an allotetraploid (2n = 4x = 52), possessing A and D genomes, has multiples of 2 COG genes composing each family except COG6 which has 3 copies [61, 85]. The results presented here show that species understood to be diploid have a single COG gene for each family member except in very few cases, a duplication. In contrast, species understood to have genome duplications and, in particular, more recent duplications have more extensively duplicated copies of its COG genes. In the examples provided here, the hexaploid *T*. *aestivum* stands out in mostly having multiples of 3 COG genes per family. As stated, more work needs to be done to understand these genes better. The results point out another interesting feature of the COG genes that appears to be the maintenance of a fixed number of COG genes in relation to the different gene families in the diploid species where at least the maintenance of gene duplication events appears not to be occurring. In contrast, more complicated features of the COG genes that relate to their transcription (alternate splicing) and possible protein functions, in particular in the polyploid species, have been determined and will be addressed in the next section.

### Localized COG gene duplication is uncommon

The results show that localized, tandem duplication of the COG genes is uncommon. Notably, *G*. *max* did not experience tandem duplication of its COG genes as observed for some of its other defense genes [36]. However, *M*. *esculenta* had tandemly duplicated COG4. *G*. *hirsutum* had tandem duplication of COG6. *S*. *lycopersicon* COG3 was tandemly duplicated. The nature of these tandem duplications remains unclear.

### Alternate splicing of COG genes

**Fig 5:**
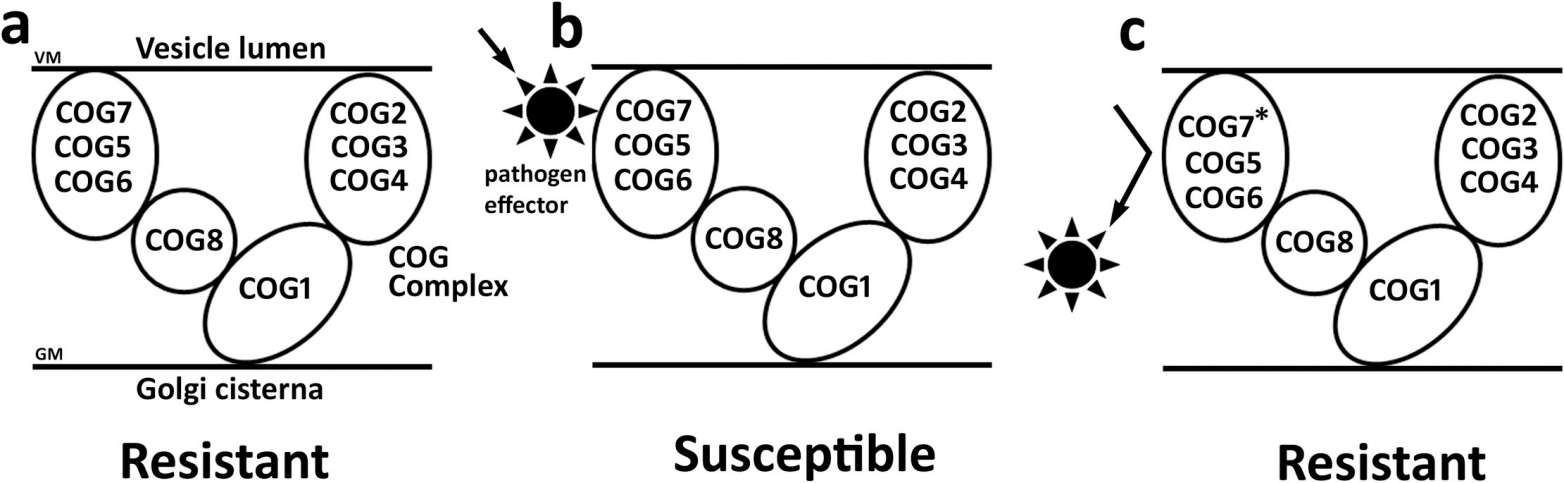
Model. A. The COG complex functions in defense. **B**. A pathogen effector alters the functionality of the COG complex, leading to susceptibility. **C**. The COG complex composition becomes altered with a splice variant (COG7-2-b*, Glyma.12G013000.2) which alters the ability of the pathogen effector to bind, restoring the ability of the COG complex to function in defense, leading to a resistant reaction. VM, vesicle membrane; GM, Golgi membrane. The position of the COG proteins in relation to the Golgi and vesicle membranes does not imply specific interactions.

The results presented here so far have focused in on identifying the numbers of COG genes in the studied plants. During the course of the proteomic studies, a number of COG protein products that are the outcomes of possible RNA splicing have also been identified. For example *A*. *thaliana* COG1 has 4 alternate splice variants AT5G16300.1, AT5G16300.2, AT5G16300.3 and AT5G16300.4. Expression experiments for AT5G16300.1 show it is expressed in low quantities in leaves treated with ammonia, leaves treated with urea, and high in roots treated with nitrate [49]. Unfortunately, the RNA seq experiments did not differentiate between the transcript abundance for the different splice variants. Alternative splicing is known to occur as a stress response and reaction to various stimuli with experiments showing that in *A*. *thaliana* 22–30% of intron-containing genes undergo alternative splicing [116–118]. Mutants of COG8 impair proper splicing in humans, leading to congenital disease [119]. Recent experiments performed in *A*. *thaliana* show that MAPK3, MAPK4 and MAPK6 regulate pathogen activated molecular pattern (PAMP)-induced differentially alternative spliced events through alternative splicing of splicing factors, themselves, and protein kinases that are related to immunity are altered [120]. The *G*. *max* MAPK3, 4 and 6 all function in its defense response to *H*. *glycines* parasitism [32]. From these studies, a model by which the splice variants could function in the defense process is presented (**Fig 5**). Pathogen effectors capable of binding to *G*. *max* membrane fusion proteins that function in defense have been identified [121]. Consequently, the impairment of binding through changes in the primary structure of the plant protein occurring through alternate splicing of its mRNA could occur.

Transcriptomic data has been useful in identifying genes that function in defense in other plant pathosystems. The analyses presented here have extracted data on splice variants of important crop species to the U.S. and globally. In most cases, the number of variants is low or have not been adequately studied or confirmed by RNA seq analyses. For example in *B*. *vulgaris*, only a single transcript type has been identified for each COG gene. It is possible that the *B*. *vulgaris* genome has not been examined extensively enough to identify all of its COG gene RNA splice variants since even reduced genomes like *A*. *thaliana* have multiple splice variants. In the most extensive example of COG gene splice variants identified in the analysis presented here, *H*. *vulgare* HORVU7Hr1G107700 (COG7) has 31 different splice variants. Splice variants perform important functions in plants and alternate splicing occurs during defense to pathogen attack [122]. The diversity of COG gene splice variants could be an important feature to consider when examining the role pathogen effectors may have on plant multiprotein complexes [121, 123, 124]. COG7-2-b appears to be a COG gene that is typically expressed to low levels, consistent with it not being the primary transcript. However, its expression occurs in a specialized cell type (syncytia) undergoing a defense response. Consequently, perhaps COG7-2-b has a function that is important to the defense response, explaining why it is otherwise expressed at low levels in whole roots. A similar expression profile is shown for COG8-1-a which functions in defense as compared to COG8-1-b. The results demonstrate that there is much left to be learned regarding the splice variants and their biological function(s).

### COG genes exhibit some co-regulated expression

Analyses reveal that α-SNAP and SYP38 expression level influences each other’s relative transcript abundance as revealed in overexpression and RNAi experiments [30]. This concept has been further examined in other SNARE components that function in defense in the *H*. *glycines* pathosystem [38]. The experiments presented here reveal that several COG components also exhibit co-regulated gene expression. The results expand on the knowledge of genes functioning in the defense in the *G*. *max*-*H*. *glycines* pathosystem that experience co-regulation. Related experiments show that MAPKs functioning during defense in this pathosystem also are co-regulated [32]. MAPKs, as an important signaling platform, could be expected to greatly influence gene expression occurring during biotic stress [32].

### COG gene expression is influenced by MAPK expression

McNeece et al. (2019) [32] performed a functional transgenic analysis of the 32 members of the *G*. *max* MAPK gene family, finding that 9 of them function in its defense response to *H*. *glycines* parasitism. Subsequent RNA seq analyses of RNA isolated from the 9 defense MAPK-OE roots have been performed [51]. Analyses of the RNA seq data presented here has led to the identification that some cases, *G*. *max* COG gene expression is influenced by the defense MAPKs. The results have been confirmed by RT-qPCR analyses. The genetic analyses of McNeece et al. (2019) [32] have determined that *NON-RACE-SPECIFIC DISEASE RESISTANCE1* (*NDR1*) and *BOTRYTIS INDUCED KINASE1* (*BIK1*), functioning in ETI and PTI, respectively, converge on the MAPK network to induce the transcription of defense genes that themselves have been proven to function in the defense process. More recent experiments presented by Klink et al. (2021) [25] have shown *G*. *max BRI1-ASSOCIATED RECEPTOR KINASE 1* (*BAK1*) overexpression increases MAPK3 transcript anundance while *BAK1* RNAi decreases MAPK3 transcript abundance as compared to controls. Furthermore, *G*. *max BAK1* overexpression decreases *H*. *glycines* parasitism by 67% while *BAK1* RNAi increases it by 4.9 fold as compared to controls [25]. The result implies specific pathogen recognition receptors alone or in combination play important roles in the defense response that *G*. *max* has to *H*. *glycines* [25]. The result is consistent with the involvement of the BAK1-interacting cytoplasmic kinase BIK1 in the *G*. *max* defense response to *H*. *glycines* parasitism [30]. The experiments presented here reveal that those MAPK-induced genes include COG complex genes that function in in *G*. *max* during its defense response to *H*. *glycines*. These observations have broad implications. The results presented here may aid in the determination of genetic platforms that underlie defense in other important crop plants [125, 126]. An interesting finding from these results is the identification that MAPK-OE samples, including MAPK2, MAPK3-1, MAPK3-2, MAPK4, MAPK5-3, MAPK6-2, and MAPK20-2, measure induced levels of expression of COG1-2. COG1 binds to the Golgi face and is the point of attachment for the other COG proteins of the A and B subcomplexes [1, 3–6]. Another interesting observation is the induced expression of COG7-2-b in the MAPK2, MAPK3-1 and MAPK6-2 overexpression lines, MAPKs that are important defense components in other plant systems. COG7 binds to the vesicle surface. Consequently, it is possible that having a sufficient amount of the point of attachment for vesicles (COG7) is an important for functionality under certain circumstances like plant defense.

The *G*. *max* COG gene family has already been presented and, unlike the diploid *A*. *thaliana*, has two paralogs of each gene which is consistent with its allotetrapoid nature [17, 62]. Polyploidization plays an important role in plant evolution and in particular, many agricultural crops are known to be polyploid in nature [127, 128]. The analysis presented here shows that the crop plants under study here exhibit various levels of duplication of their COG gene family members. In addition to this gene duplication, there appears to exist multiple splice variants for each of the COG genes. The *G*. *max* splice variant COG7-2-b which functions in defense to *H*. *glycines*, appears to exhibit that alternative splice variants are expressed in specific cell types at certain times of a defense response and are important to the defense process [17]. The results may have important implications for understanding basic aspects of the COG gene families of the other studied significant crop plants presented here including *M*. *esculenta*, *Z*. *mays*, *O*. *sativa*, *T*. *aestivum*, *H*. *vulgare*, *S*. *bicolor*, *B*. *rapa*, *E*. *guineensis*, *S*. *officinalis*, *S*. *tuberosum*, *S*. *lycopersicum*, *G*. *hirsutum* and *B*. *vulgaris*. In humans, a mutation in a splice site of COG8 results in a congenital disease, demonstrating that proper splicing of COG genes relates to their functionality [119]. Experiments aimed at understanding the diversity of these alternate splice variants and the regulation of their expression, in particular, using cell-type specific procedures should provide important insight and a tool to understand their biological role(s).

## Supporting information

S1 Table RT-qPCR primers used in the analysis. (XLSX)

S2 Table Gene expression of control and syncytium cells relating to Fig 2. (XLSX)

S3 Table COG gene information for *A*. *thaliana*. (XLSX)

S4 Table COG gene information for *G*. *max*. (XLSX)

S5 Table COG gene information for *M*. *esculenta*. (XLSX)

S6 Table COG gene information for *Z*. *mays*. (XLSX)

S7 Table COG gene information for *O*. *sativa*. (XLSX)

S8 Table COG gene information for *T*. *aestivum*. (XLSX)

S9 Table COG gene information for *H*. *vulgare*. (XLSX)

S10 Table COG gene information for *S*. *bicolor*. (XLSX)

S11 Table COG gene information for *B*. *rapa*. (XLSX)

S12 Table COG gene information for *E*. *guineensis*. (XLSX)

S13 Table COG gene information for *S*. *officinalis*. (XLSX)

S14 Table COG gene information for *B*. *vulgaris*. (XLSX)

S15 Table COG gene information for *S*. *lycopersicon*. (XLSX)

S16 Table COG gene information for *S*. *tuberosum*. (XLSX)

S17 Table COG gene information for *G*. *hirsutum*. (XLSX)

S18 Table COG protein motifs for selected plant species used in the analyses, including *G*. *max*, *B*. *rapa*, *G*. *hirsutum*, *M*. *esculenta*, *E*. *guineensis*, *S*. *tuberosum*, *S*. *officinalis* and *Z*. *mays*. Only plants with duplicated COG genes are analysed for the purpose of identifying protein domains that may be indicative of neofunctionalization of paralogs. Details of PFam (PF) families can be determined at https://pfam.xfam.org/.
(XLSX)

S19 Table COG gene expression data for alternative splice variants not expressed in any of the *G*. *max* MAPK-OE roots, but whose expression data is obtained from Phytomine from leaf, nodule, pod, root, root hair, seed, SAM, and stem (Goodstein et al. 2012). (XLSX)
